# Mitral Valve Repair for the Treatment of Acute Bacterial Endocarditis: Analysis of a 10-Year Single-Center Experience

**DOI:** 10.3390/jcm14227907

**Published:** 2025-11-07

**Authors:** Martina Musto, Sonia Lerta, Gloria Sangaletti, Raffaele Bruno, Elena Seminari, Giulia Magrini, Romina Frassica, Monica Wu, Stefano Pelenghi, Pasquale Totaro

**Affiliations:** 1 Department of Medicine, Surgical, Diagnostic and Pediatric Science, University of Pavia, 27100 Pavia, PV, Italy; martina.musto01@universitadipavia.it (M.M.); sonia.lerta01@universitadipavia.it (S.L.); gloria.sangaletti01@universitadipavia.it (G.S.); raffaele.bruno@unipv.it (R.B.); 2 Cardiac Surgery Unit, IRCCS Foundation Hospital “San Matteo”, Piazzale Golgi, 27100 Pavia, PV, Italy; s.pelenghi@smatteo.pv.it; 3 Infectious Diseases Unit, IRCCS Foundation Hospital “San Matteo”, 27100 Pavia, PV, Italy; e.seminari@smatteo.pv.it; 4 Cardiology Unit, IRCCS Foundation Hospital “San Matteo”, 27100 Pavia, PV, Italy; g.magrini@smatteo.pv.it (G.M.); r.frassica@smatteo.pv.it (R.F.); m.wu@smatteo.pv.it (M.W.)

**Keywords:** mitral valve repair, mitral valve replacement, acute bacterial endocarditis, surgical outcomes in cardiac surgery

## Abstract

**Background/Objectives:** Acute bacterial endocarditis (ABE) is a frequent situation and continues to be a challenge. Mitral valve involvement during acute bacterial endocarditis is often the result of the spread of the endocarditic process from the adjacent aortic valve. Mitral involvement, on the other hand, could also be an expression of the initial localization of the bacteria. The best option for treating mitral ABE is still a matter of debate. Recent reports have shown satisfactory results with mitral reconstructive techniques in the treatment of mitral ABE. In this study, we present a comprehensive review of our 10-year institutional experience in the surgical management of acute mitral endocarditis with a focus on technical considerations, outcomes, and the durability of mitral valve repair in this high-risk population. **Methods:** We queried the institutional database, cross-referencing patients admitted with a diagnosis of “acute bacterial endocarditis” with patients undergoing surgical procedures for “valvular disease” at our division. Out of 1136 valvular procedures listed in our PACS database, 180 patients were admitted with a diagnosis of active acute endocarditis, and 46 included treatment of the mitral valve. We analyzed and compared short- and long-term follow-up (ranging from 3 to 141 months with a mean of 42 ± 38 months) of these 46 patients, dividing them into two groups: mitral valve repair (MVr) and mitral valve replacement (MVR). **Results:** 18 (40%) patients underwent reconstructive treatment of the mitral valve, and 28 (60%) underwent mitral valve replacement. Cumulative in-hospital mortality was 10% (5 pts, all from the MVR group), however, with no difference between the two groups. A shorter time gap from diagnosis to surgery (<10 days) was the only predictive factor for early mortality. A further 11 patients died during follow-up (2 from group A and 9 from group B). Long-term survival, on the other hand, was negatively influenced by MV surgical replacement (*p* = 0.0178), older patients’ age (>60 years), and urgent surgical procedures. Finally, patients with MVr also experienced a favorable postoperative event-free curve for endocarditis recurrence *(p* = 0.0260*)* and time elapsed before recurrence (*p* = 0.0438). **Conclusions:** Mitral valve repair in the case of active endocarditis could be a treatment associated with more favorable outcomes, providing that a complete eradication of infective tissue can be accomplished. Conservative treatment, when feasible, seems to offer favorable cumulative long-term outcomes.

## 1. Introduction

Despite advances in diagnostics and therapeutics, acute bacterial endocarditis (ABE) remains one of the most serious challenges in contemporary medicine. The annual incidence of infective endocarditis (IE) is estimated at 3–14 cases per 100,000 subjects/year in developed countries, and has been gradually increasing in recent years [1,2]. Aging populations, the increased use of intracardiac devices, and rising rates of intravenous (IV) drug use appear to be significant causes of such an increase. In addition, an increase in mortality has recently been reported, especially in younger patients (aged 25–44 years [3]). The pathophysiology of ABE is characterized by a microbial colonization of the endocardium, typically targeting native or prosthetic heart valves. Microbial colonization can lead to the formation of vegetations composed of fibrin, platelets, and microbial organisms. Alternatively, it may result in the erosion of the valve’s leaflets. Depending on the pathophysiological effects, surgical treatment is indicated when there is an increased risk of systemic embolization or a severe degree of valve dysfunction with relevant hemodynamic impact [4,5,6,7,8,9]. In the case of left-sided bivalvular endocarditis, the mitral valve is frequently affected by ABE following initial involvement of the aortic valve due to their anatomical proximity and shared fibrous continuity [10,11,12]. However, double valve endocarditis is reported in only 15–20% of ABE cases, and mitral involvement is often the primary localization of ABE (usually in patients with a known mitral valve prolapse) [13,14,15]. In the case of mitral valve involvement, the surgical indication depends on the general concepts previously summarized: active infective status despite antibiotics, high risk of embolization of vegetation, and severe valve dysfunction with relevant hemodynamic impact. Once surgery is indicated, the choice between mitral valve repair (MVr) and replacement (MVR) remains a matter of debate, although both the European Society of Cardiology (ESC) and the American Association for Thoracic Surgery (AATS) advocate for repair over replacement when technically feasible [7,8]. Extensive reviews have reported a valve repair rate between 33 and 86% and seem to suggest better long-term outcomes, including preservation of left ventricular function, lower risk of prosthesis-related complications, and improved survival rates [16,17,18,19,20,21,22] following conservative surgical treatment. On the other hand, conflicting results have also been reported in terms of incidence of reoperation following MVr [19,20]. Over the past 10 years, our hospital has established a close multidisciplinary collaboration focused on the diagnosis, assessment, and treatment (both medical and surgical) of bacterial endocarditis, thus anticipating the call for the establishment of an “endocarditis team” included in the latest guidelines. In this paper, we analyze and summarize the results of the first decade of activity of our “endocarditis working group”, focusing on the surgical management of acute mitral endocarditis, particularly on the technical considerations, outcomes, and durability of mitral valve repair in this high-risk population.

## 2. Materials and Methods

This is a single-center, retrospective, observational study based on our experience in the last decade (from November 2013 to November 2024) on the surgical treatment of acute bacterial endocarditis involving a native/prosthetic mitral valve. Patients were included in the study by cross-referencing data from two institutional databases: (a) the institutional database “ORMAWEB,” a registry compiled by cardiac surgeons that collects all individual surgical procedures performed under the care of the Cardiac Surgery Division; (b) the institutional endocarditis registry “STEADY”, a registry compiled by colleagues in the Infectious Diseases Unit that collects all patients admitted to our hospital with a diagnosis of “acute bacterial endocarditis”. Our hospital being an interregional hub for infectious diseases, the STEADY registry includes a large number of patients referred from peripheral hospitals following a diagnosis of endocarditis. More specifically, all patients undergoing mitral valve surgery were extracted from the ORMAWEB database. The total number of patients was then filtered by entering patients with a preoperative diagnosis of “bacterial endocarditis” into an advanced search. The data extracted from the advanced search were then cross-referenced with data from the “STEADY” registry to confirm the preoperative diagnosis and extract additional preoperative data. Patients with confirmed diagnoses in the two databases were included in the study and all extracted data were entered into the appropriate dataset. Overall, 594 patients with a surgical procedure involving the mitral valve were registered on the operative database, 180 with a diagnosis of active bacterial endocarditis (16%). Out of these 180 procedures, 46 (26%) included surgical intervention on the mitral valve, whether it was valve repair or replacement. The study was conducted in accordance with the Declaration of Helsinki, and approved by the Institutional Ethics Committee (protocol number P-2020006). All patients signed informal consent to both surgical procedure and anonymous data publications. The medical history and clinical and laboratory data of all patients were prospectively entered into the institutional database at the time of admission and then analyzed retrospectively for the purposes of this study. Early postoperative outcomes and in-hospital (30 days) mortality were considered as primary endpoints and were compared within two study groups: patients undergoing mitral valve repair (group A, MVr) and those undergoing mitral valve replacement (group B, MVR). As secondary endpoints, we considered all events reported during follow-up (following patient discharge from hospital), including postoperative re-admission, recurrence of endocarditis, re-operation rate, and long-term mortality. Secondary endpoints were also compared between the two study groups. Follow-up was 100% completed by querying the institutional “Fenix” database, which includes all patients’ attendance to our hospital’s outpatient clinics. For patients lost to follow-up due to failure to attend a follow-up visit, confirmation of any death was finally obtained by querying the regional health registry.

## 3. Statistical Analysis

All data were recorded in a designed database and statistical analysis was performed using Medcalc software (Medcalc^®^ version 8.1.1.0; Acacialaan 22, 8400 Ostend-Belgium). Normal distribution for continuous variable was tested using the Kolmogorov–Smirnov and D’Agostino tests to optimize further statistical test choice. Data were expressed as mean ± standard deviation (SD) or median (with 95% CI), according to the results of the normal distribution test. Comparative statistics for continuous variables was performed, depending on the normal distribution, using unequal variance Mann–Whitney/Kruscal–Wallis tests. A The comparison of categorical variable estimation was obtained with a chi-square analysis (Fisher’s exact test when appropriate) or a Mann–Whitney test. Predictive risk factors for early mortality were analyzed at univariate analysis and variables with *p* < 0.20 were entered in the multivariable model (see Appendix A for a list of variables and detailed results). A *p*-value of 0.05 was considered statistically significant. A log-rank test for a Kaplan–Maier curve was used to evaluate late survival and the impact of surgical procedure. Cox proportional hazard regression was used to analyze the impact of several parameters in long-term survival.

## 4. Results

Forty-six cumulative patients who underwent surgical treatment for isolated acute mitral infective endocarditis (24 pts, 52%) or bivalvular acute endocarditis (22 pts, 48%) were included in the study. Eighteen patients (39%) underwent reconstructive mitral valve surgery and were therefore enrolled in **group A (MVr)**; on the other hand, twenty-eight patients (61%) underwent mitral valve replacement and were therefore enrolled in **group B (MVR)**. As summarized in Table 1, patients’ gender and incidence of bivalvular involvement did not differ between the two groups. Patients’ age, however, significantly differed between group A and group B in terms of both mean age (*p* = 0.0143) and the incidence of patients older than 60 years (*p* = 0.0367).

Looking at the time interval between diagnosis and surgery, as well as the time elapse of the infection (time required to obtain positive blood cultures and time required to obtain negative blood cultures), as summarized in Table 1, no significant differences were found between the two groups.

**Figure 1:**
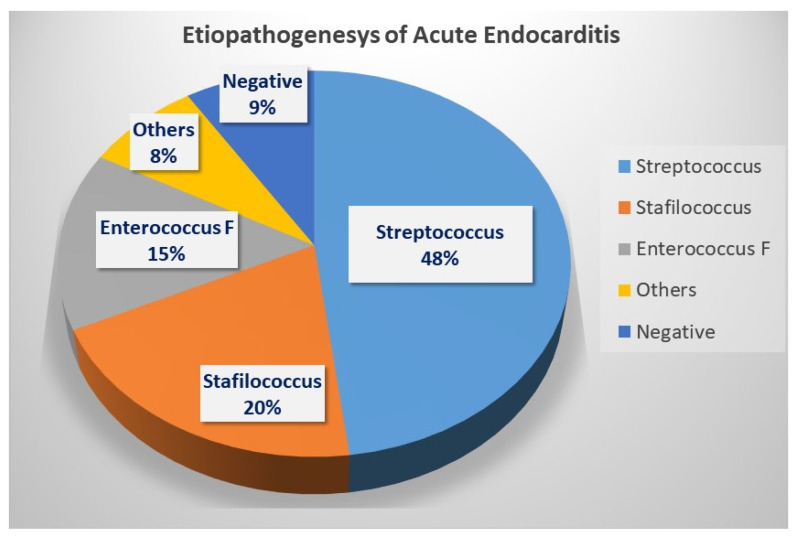
Breakdown of bacterial isolation.

The bacterial agent responsible for acute endocarditis was clearly identified in 40 patients (87%), while in 2 patients (4%) it remained unknown (patients transferred from another hospital with a diagnosis of endocarditis based on a preliminary identification of germs in culture, but without subsequent identification). Furthermore, in four patients (9%), the blood cultures were negative throughout the entire hospital stay. The detailed breakdown of bacterial isolation is shown in Figure 1.

Regarding surgical techniques, within the MV repair group (group A), a variety of surgical techniques were employed, often in combination. Leaflet resection was the most commonly used technique (eight patients, 42%), followed by direct leaflet repair (six patients, 32%), and leaflet plication (three patients, 16%). Additional procedures, including patch reconstruction or chordal shortening, were performed in two patients (10%). Mitral annuloplasty was performed in the majority of cases (16 patients, 84%), underlining its pivotal role in restoring valvular competence and geometry. In group B, 16 patients (57%) received a biological prosthesis and 12 patients (43%) received a mechanical prosthesis. Out of the 22 patients who underwent double valves surgery, the majority received aortic valve replacement as a combined procedure (15 patients, 68%), with no difference between the two groups. Timing of surgery was also not different between the two groups (Table 2).

**Figure 2:**
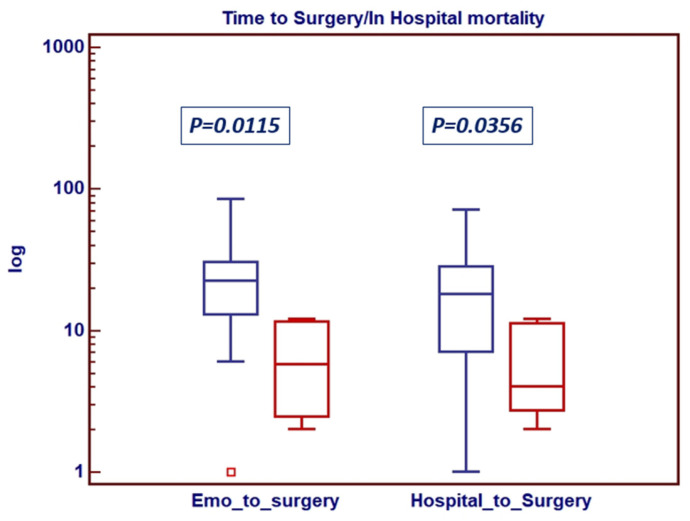
Determinants of in-hospital mortality. Box-and-whiskers dot plots showing univariate relationship between log-based time from positive blood culture and in-hospital admission to surgical procedure and in-hospital death. *Borders of box: 1st and 3rd quartile; line in the box: median; whiskers: maximum and minimum values of non-outliers. Error bars represent 95% CI for median. p-value calculated by means of Mann–Whitney test*.

By looking at the primary endpoints of the study, it can be gathered that the overall in-hospital mortality across the cohort approached nearly 10% (5 pts, 10.8%), all from group B (18% in-hospital mortality for group B), but with no statistically significant difference between the two groups (*p* = 0.1143). Univariate analysis indicated a shorter time from positive blood cultures and hospital admission to surgery as the only predictive factor for in-hospital mortality (Figure 2).

**Figure 3:**
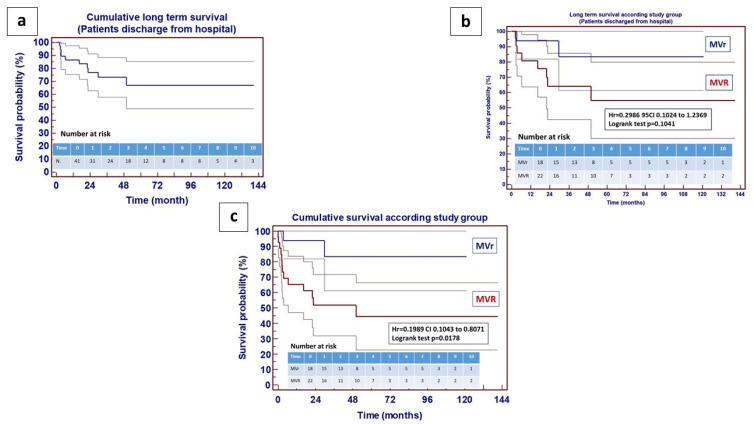
Long-term survival following surgery for active endocarditis. (**a/left**): cumulative long-term survival in patients discharged from hospital—95% CI; (**b/right**): long-term survival in patients discharged from hospital according to study group; (**c/bottom**): overall long-term survival including all patients enrolled in the study according to study group—95% CI; number at risk stratified per year.

Age and etiology of acute endocarditis did not impact early mortality. Furthermore, multivariate analysis did not confirm any predictive factor for in-hospital mortality (see Appendix A for a list of variables and detailed results). Furthermore, as a final comparison regarding the primary endpoints, the analysis of early postoperative parameters did not reveal any significant differences between the two groups (Table 2). Moving to the secondary endpoints, within patients discharged home following operation, a further 11 patients (27%) were lost at follow-up, 2 from group A (11%) and 9 from group B (40%). Follow-up ranged from 3 to 141 months (mean 42 ± 38 months) with no difference between the two groups (*p* = 0.4806). Cumulative long-term survival is shown in Figure 3a. Analysis of the Kaplan–Meier curve, in patients discharged alive from hospital, confirmed the trend towards better survival in group A, however, with no statistical significance in the log-rank test (Figure 3b). The overall survival curve (including all patients enrolled in the study), on the other hand, showed a significant difference favorable to patients who underwent MVr (Figure 3c).

**Figure 4:**
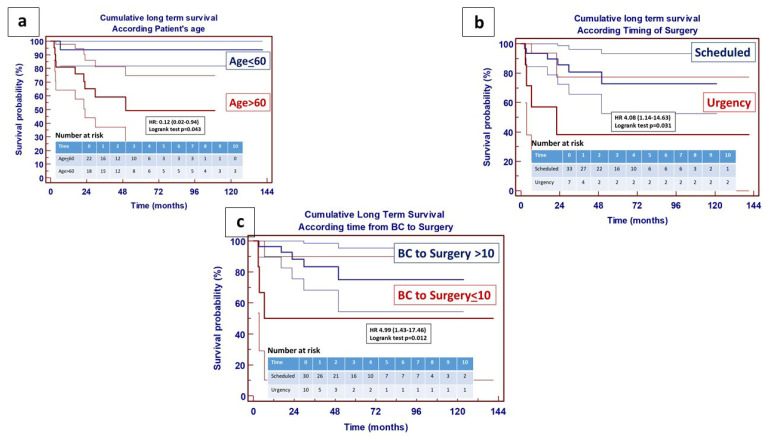
Determinants of impaired long-term survival following surgery for active endocarditis. (**a/left**): long-term survival in patients discharged from hospital according to age > 60—95% CI; (**b/right**): long-term survival in patients discharged from hospital according to timing of surgery—95% CI; (**c/bottom**): long-term survival in patients discharged from hospital according to time from positive blood culture to surgery—95% CI. Number at risk stratified per year.

Cox regression hazard identified, within a continuous variable, two significant factors for poor early survival: patients’ age (*p* = 0.0231) and lower time gap from positive blood culture to surgery (*p* = 0.0234). Patient’s age > 60 years, urgent timing of surgery and performing surgery within 10 days from a positive blood culture, furthermore, significantly impacted cumulative long-term survival among patients discharged from hospital (Figure 4). Etiology of acute endocarditis, on the other hands, did not seem to impact the long-term survival curve (*p* = 0.3524).

**Figure 5:**
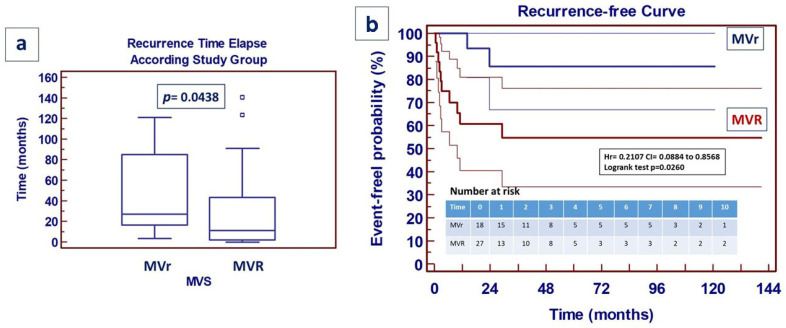
Recurrence of endocarditis following surgically treated acute mitral endocarditis. (**a/left**): box-and-whiskers dot plots showing univariate relationship between time elapse of endocarditis recurrence and study groups. (*Borders of box: 1st and 3rd quartile; line in the box: median; whiskers: maximum and minimum values of non-outliers. Error bars represent 95%CI for median). p-value calculated by mean of Mann–Whitney test). (***b/right**): Recurrence of acute endocarditis event-free curve—95%. *Number at risk stratified per year*.

Looking at the incidence of postoperative unfavorable events, overall, 12 patients (26%) experienced recurrence of endocarditis (with positive blood culture), 10 (36%) from the VVR group and 2 (11%) from the MVr group (OR: 4.4 95% CI 0.84 to 23.89; RR 1.38 CI 1.00 to 1.92). Both the time elapse analysis (Figure 5a) and the event-free curve (Figure 5b) show a significant difference between the two groups. Overall, four patients (8.3%), underwent reoperation, one (5%) from group A and three (11%) from group B (OR: 2.04; 95% CI = 0.19 to 21.29; RR: 1.05 95% CI: 0.89 to 1.25) without a significant difference in the event-free curve.

**Figure 6:**
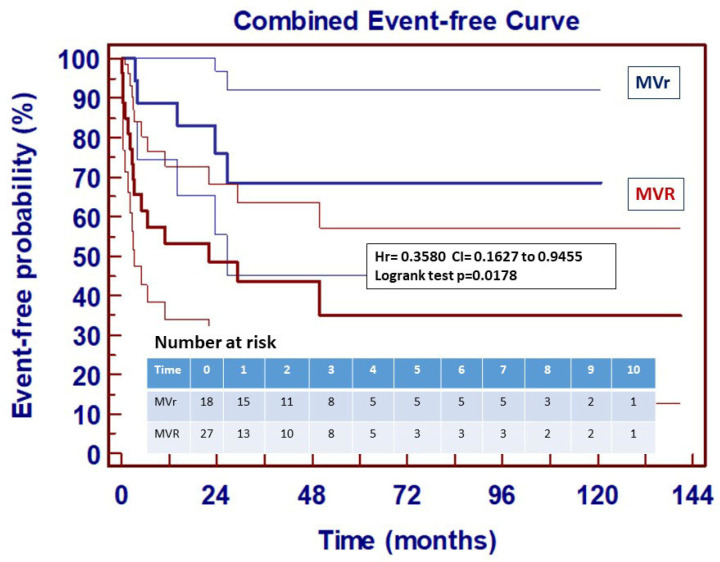
Event-free curve of combined unfavorable events (death, recurrence of endocarditis, reoperation) following surgically treated acute mitral endocarditis—95% CI.

Finally, an event-free curve (Figure 6) for combined unfavorable events shows a significant difference between the two groups.

## 5. Discussion

In this study, we addressed the very relevant and current problem of the outcomes of patients undergoing surgical treatment of acute mitral endocarditis. We therefore analyzed our single-center experience over the last decade in treating this patient population. We focused on the analysis of short- and long-term outcomes and compared the results between two surgical strategies: conservative and replacement. In the 1990s, the presence of mitral infective endocarditis in itself was considered as a contraindication to mitral valve repair until it was suggested by the group of Dreyfus [23]. However, recently, the conservative approach, when feasible, has been recognized as a valid and often favorable alternative [16,17,18,20,21,22]. Despite this, it appears that the percentage of acute mitral endocarditis with surgical repair rather than prosthetic mitral valve replacement remains below 50% [20,21]. Current ESC 2023 guidelines reported that “it cannot be concluded that mitral valve repair is superior to replacement due to the high probability of ‘selection bias’” [8]. Despite recent reports of an increased percentage of reconstructive mitral valve surgery in the case of acute bacterial endocarditis, the key points for the choice of reconstructive strategy have not been clearly elucidated yet [5,16,19,20,21]. Given the retrospective nature of our study, the reason for the choice of surgical technique could not be easily identified, and therefore we investigated peculiar characteristics that could have driven the decision. We hypothesized that the decision-making process regarding repair or replacement could be influenced by the preoperative condition of the patients, intraoperative findings, and technical complexity, particularly the surgeon’s qualitative assessment of the valve damage [19]. Other factors, such as the complexity of clinical cases, late diagnosis, and delayed referral for surgery may also influence the decision in favor of valve replacement, as suggested elsewhere [24]. Our experience, however, failed to clearly indicate significant differences in preoperative characteristics between the two groups, except patients’ age (younger for patients undergoing mitral valve repair), a finding that is consistent with previous experiences [25,26,27]. Patients’ age, therefore, appears to be the main factor in the choice of surgical procedures. Since the younger patient group also had a higher percentage of patients with a history of intravenous drug abuse, a close correlation between this factor and the choice of mitral repair technique could be hypothesized [28]. On the other hand, extensive single leaflet destruction, annular abscesses, leaflets perforations, or bivalvular involvement do not necessarily preclude the feasibility of valve repair, particularly when performed by experienced surgeons [21]. Advanced reconstructive techniques and aggressive debridement can enable effective repair while achieving radical control of infection. Analysis of our primary endpoints appears to demonstrate good early postoperative outcomes in patients undergoing mitral valve reconstruction compared with those undergoing mitral valve replacement. Our overall in-hospital mortality is comparable to that reported in previous reports [9,29]. Although all patients who died in hospital belonged to group B, our experience did not confirm the statistically significant difference in favor of the repair strategy previously reported by other studies [19,30]. It is certainly interesting that in our study we identified a shorter interval between the diagnosis of acute bacterial endocarditis (and hospital admission) and surgery as a significant predictor of early negative outcomes. This original finding may suggest that, when possible, waiting for the completion of antibiotic therapy could be a key factor for a positive early outcome, regardless of the type of surgery and the number of valves to be treated [31,32,33]. Moving on to the secondary endpoints, our study provided additional insight. First, we were able to confirm that, despite adequate and successful treatment, acute bacterial endocarditis requiring surgical treatment still has a significant impact on the long-term survival of patients, with an overall 5-year survival rate not exceeding 70% [10,18,34]. Despite the non-significant difference in terms of hospital mortality, the cumulative analysis (which includes all patients enrolled in the study) of long-term survival showed an improvement in survival in patients undergoing MVr. A significant impact on long-term survival, on the other hand, was also confirmed for other specific conditions, such as age over 60 and the timing of surgery, when considered urgent and performed within 10 days of a positive blood culture result. The impact of young age, as mentioned in relation to the primary endpoints, is certainly not new. The evidence of the impact of the timing of surgery, on the other hand, deserves further comment and is certainly noteworthy because, in our opinion, it could be a suggestion for optimizing surgical timing to improve postoperative outcomes. In patients undergoing urgent surgery, the progression of the infection could be the cause of late complications, despite early intervention. The optimal timing for surgery in patients with infective endocarditis has been widely debated in the past, and no definitive resolution has yet been reached, as conflicting results have been reported on the correlation between early surgery and long-term outcomes [18,19,20,30,31,32,33,34,35,36]. However, improved long-term survival is not the only proven benefit of MVr, as interesting results have been obtained by analyzing other aspects of long-term follow-up. As previously reported by numerous studies, including extensive meta-analyses [18,19,20,21,22,37], the main benefits in long-term outcomes in patients undergoing MVR were related to a more favorable curve in terms of endocarditis recurrence. The reduction in the incidence of reoperations in our study warrants further investigation to try to clarify the association between endocarditis recurrence and clinical conditions at the time of recurrence. The critical clinical conditions could in fact influence the final decision to perform reoperation. We did not find a significant correlation between the etiology of acute endocarditis and endocarditis recurrence, as previously hypothesized [38], but again, this is probably related to the limited study population. In conclusion, we could emphasize that valve preservation in acute IE involving the mitral valve should only be attempted if a durable repair is anticipated and complete eradication of the infected tissue can be achieved. Reducing the risk of damaging cardiac conduction tissue, and therefore the potential need for a pacemaker implantation, and avoiding prosthetic valve implantation, appear to be the key factors for reducing the incidence of endocarditis recurrence and the need for reoperation in these patients. MV repair, therefore, appears to be an appropriate and effective option for patients with IE, especially when performed in younger patients and at an appropriate time, as interventional time has been shown to be pivotal in the short- and long-term outcome of these patients.

## 6. Limitations

This study presents the typical limitations of a retrospective study, starting with the difficulty in defining inclusion criteria for the two surgical groups. However, we sought to analyze the principles of the decision-making process in search of predictors of improved postoperative outcomes. The limited study population represents a further significant limitation, despite the advantages inherent in the homogeneous experience typical of a single-center study.

## 7. Conclusions

Mitral valve endocarditis is a potentially life-threatening condition that requires immediate treatment. Based on our experience, mitral valve repair in cases of active endocarditis could be the treatment of choice, providing that complete eradication of the infected tissue is guaranteed. Our results show that native valve repair leads to better postoperative survival and a lower risk of recurrence in long-term follow-up. However, other relevant factors should be taken into consideration when approaching the surgical treatment of IE involving the mitral valve, such as the patient’s age and the need for urgent intervention.

## Figures and Tables

**Table 1:** (**a**). Preoperative patients’ characteristics. (**b**). Time interval related to infective endocarditis.

**(a)**				
	**Overall** **n. 46**	**MVr** **N. 18**	**MVR** **n. 28**	***p***
**Gender** **Male** **Female**	35 (76) 11 (24)	12 (66) 6 (34)	23 (82) 5 (18)	0.2963
**Age** **Range** **Mean ± SD** **>60 years**	18–88 62 ± 16 27 (59)	18–79 54 ± 14 7 (39)	34–88 67 ± 13 20 (71)	***0.0143*** ***0.0367***
**Valve Involved** **Isolated Mitral** **Mitral + Aortic** **Mitral + Tricuspid**	24 (52) 14 (30) 8 (18)	11 (62) 4 (22) 3 (16)	13 (46) 10 (36) 5 (18)	0.7345
**Previous Cardiac Surgery**	4 (9)	2 (11)	2 (8)	0.5643
**(b)**				
	**Overall** **n. 46**	**MVr** **N. 18**	**MVR** **n. 28**	***p***
**Waiting Time for Surgery** **BC+ to Surgery** **Hospital to Surgery**	21 (13–28) 16 (9–21)	29 (17–40) 20 (7–31)	14 (9–28) 13 (6–21)	0.0553 0.3165
**Time of BC + (days)**	7 (4–8)	7 (6–8)	7 (3–10)	0.4493
**Time to BC + (hours)**	17 ± 9	16 ± 6	17 ± 10	0.8617

**MVr:** mitral valve repair; **MVR:** mitral valve replacement. Continuous data are expressed as mean ± SD or median (with 95% CI) according to the results of a normal distribution test. Categorical variables are expressed as a unit and percentage in brackets. **BC+:** blood culture positive.

**Table 2:** Surgical data.

	Overall n. 46	MVr n. 18	MVR n. 28	*p*
**Isolated Mitral Procedure** **Combined Procedure** **AVR** **TVS**	24 (52) 22 (48) 15 (68) 7 (32)	10 (56) 8 (44) 5 (62) 3 (38)	14 (50) 14 (50) 10 (71) 4 (29)	0.7489 0.5752
**Timing** **Scheduled** **Urgency/Emergency**	36 (78) 10 (22)	17 (94) 1 (6)	19 (68) 9 (32)	0.1003
**Postoperative Course** **Division Stay** **In-Hospital Stay**	14 ± 11 35 ± 18	12 ± 8 40 ± 14	14 ± 11 33 ± 18	0.6391 0.3341

**MVr:** mitral valve repair**; MVR:** mitral valve replacement; **AVR:** aortic valve replacement; **TVS**: tricuspid valve surgery. Continuous data are expressed as mean ± SD or median (with 95% CI), according to the results of a normal distribution test. Categorical variables are expressed as a unit and percentage in brackets.

## Data Availability

The data presented in this study are available on request from the corresponding author.

## Appendix A

Variables Included in Multivariate Analysis

**Continuous:**

- Age
- Time to Positive Blood Culture (hours)
- Time from Positive Blood Culture to Surgery (days)
- Time from In-Hospital Admission to Surgery (days)
- Postoperative Hospital Stay (days)

**Categorical:**

- Age > 60 years
- Patients’ Gender
- Bivalvular Involvement
- Etiology of Endocarditis
- Time to Positive Blood Culture (<10 h)
- Time from Positive Blood Culture to Surgery (<10 days)
- Time from In-Hospital Admission to Surgery (<10 days)
- Timing of Surgery
- Mitral Valve Repair
- Combined Surgical Procedure
